# Supplementary figures and images for: An integrative approach to protein sequence design through multiobjective optimization
Source: PLoS Comput Biol. 2024 Jul 11;20(7):e1011953. doi: 10.1371/journal.pcbi.1011953 (PMC11265717; doi:10.1371/journal.pcbi.1011953)

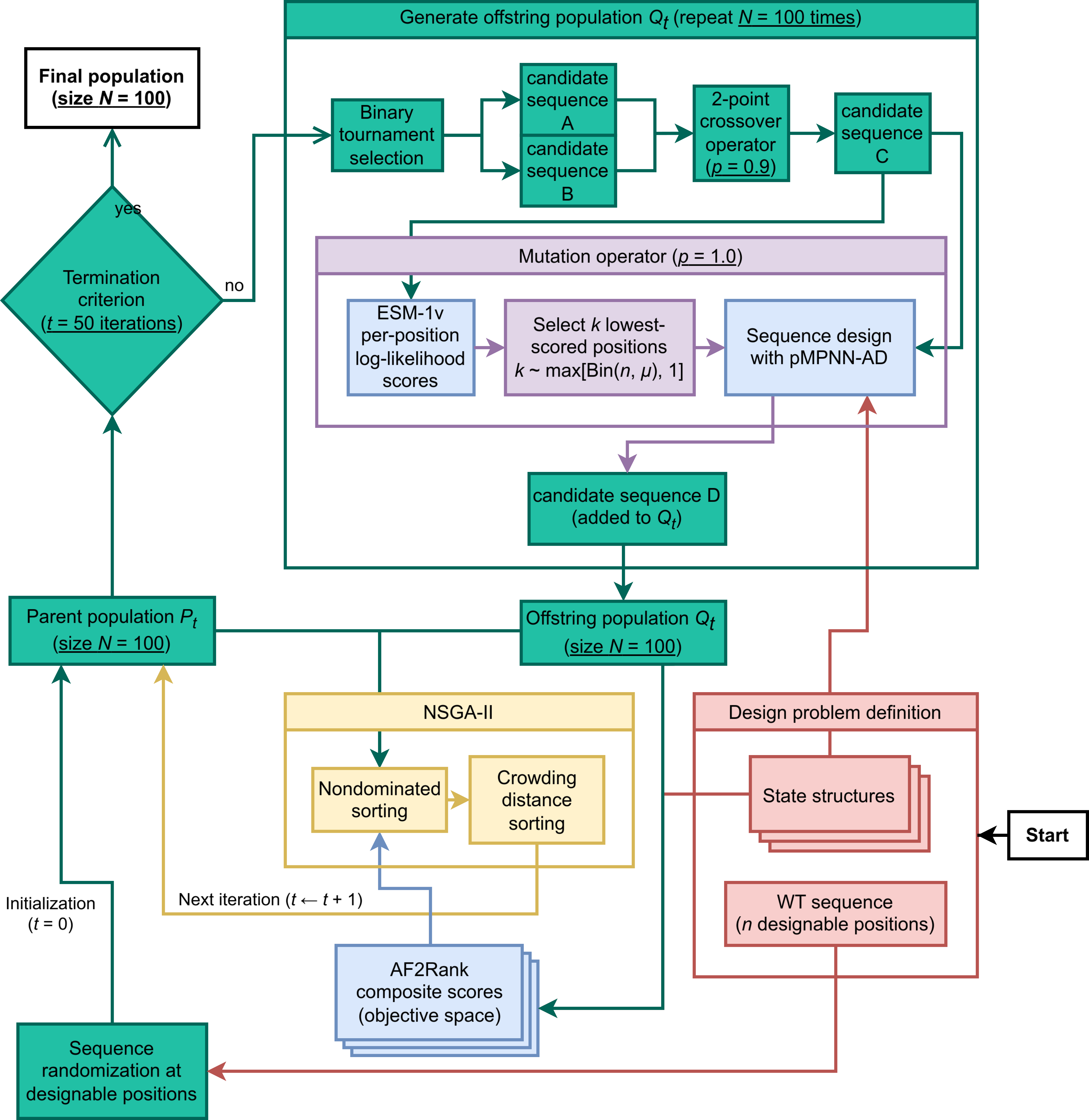

Supplement: S1 Fig — Elements of the algorithm typically found in genetic algorithms are shown in green, methods specific to Non-dominated Sorting Genetic Algorithm II (NSGA-II) are highlighted in yellow, the machine-learning models are highlighted in blue, the modifications to the mutation operator proposed in this work are highlighted in purple, and problem-specific elements are highlighted in red. The hyperparameters to the genetic algorithm and their values used in this work are shown with underlines (for the crossover and mutation operators, p is the probability that the operator is applied). See Methods for additional details. (PNG) [file pcbi.1011953.s001.png]

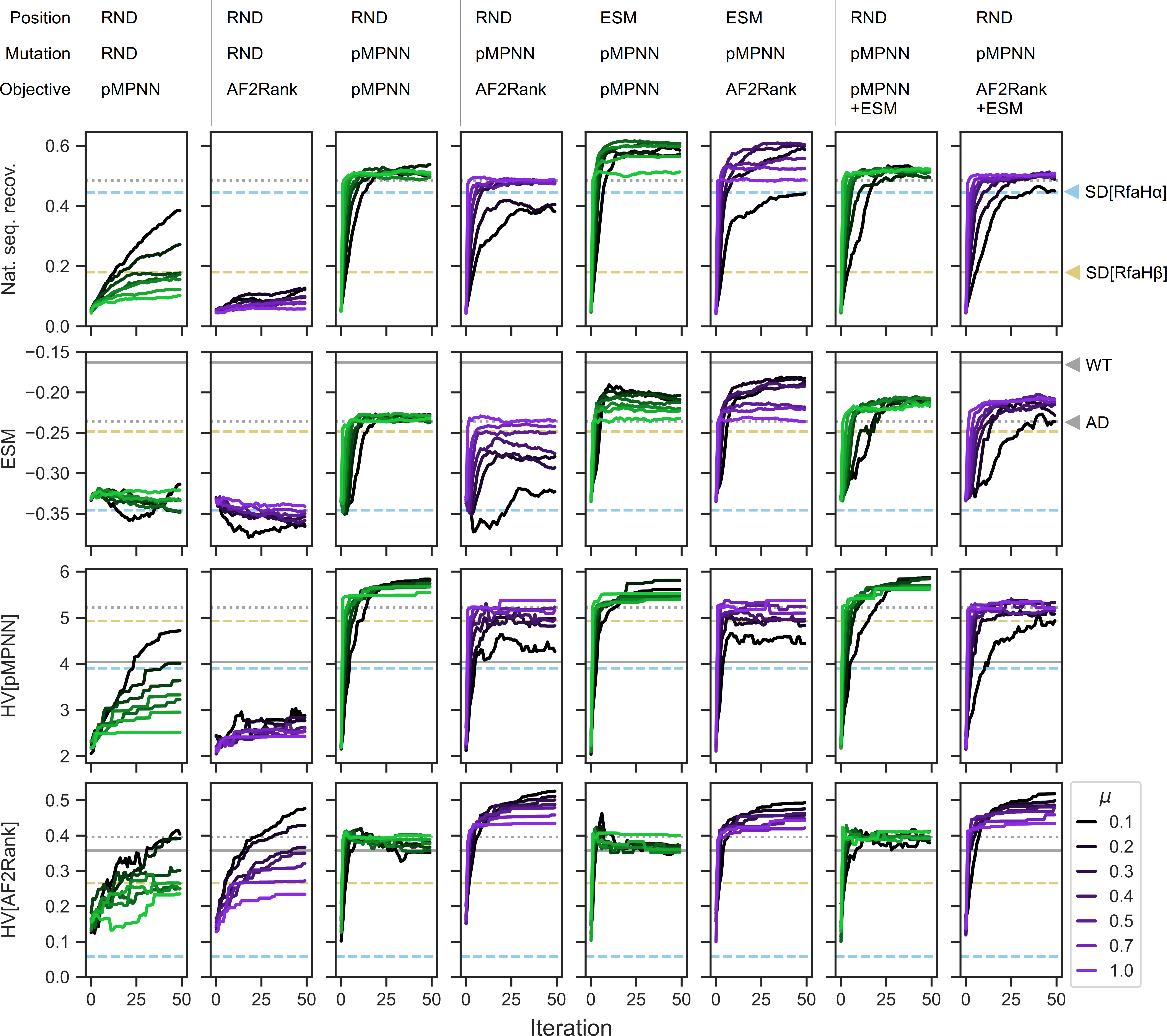

Supplement: S2 Fig — Refer to Fig 2 legend for more details. (PNG) [file pcbi.1011953.s002.png]

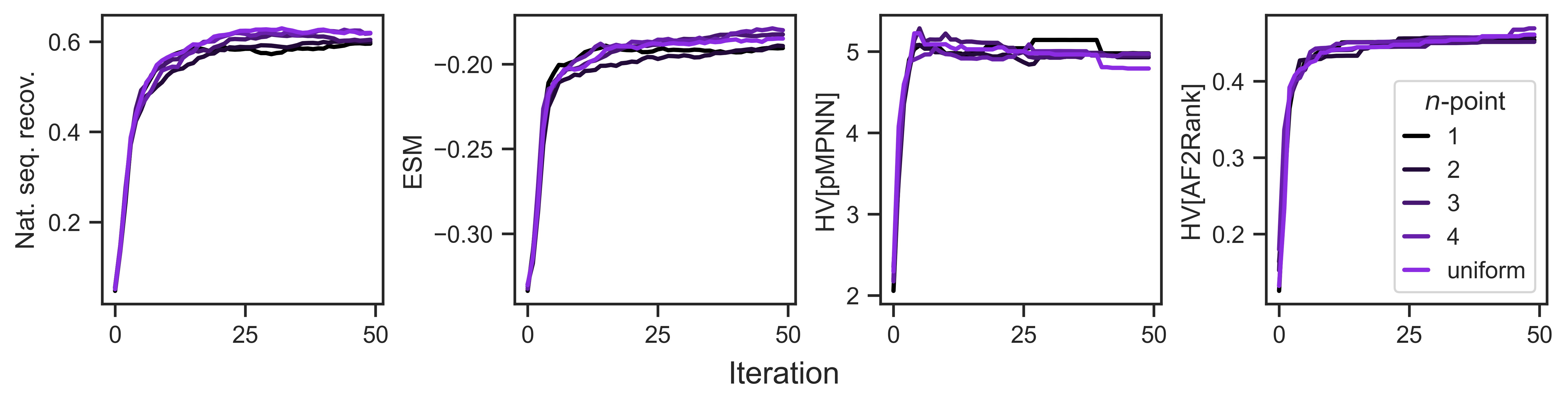

Supplement: S3 Fig — Five sets of simulations are performed with the GA[ESM,pMPNN,AF2Rank;μ = 0.3] setup, while varying the number of crossover points. For the uniform crossover operator, the residue at each designable position is chosen from either parental sequences with equal probability; effectively, for L designable positions, the uniform crossover operator is analogous to the (L−1)-point crossover operator. (PNG) [file pcbi.1011953.s003.png]

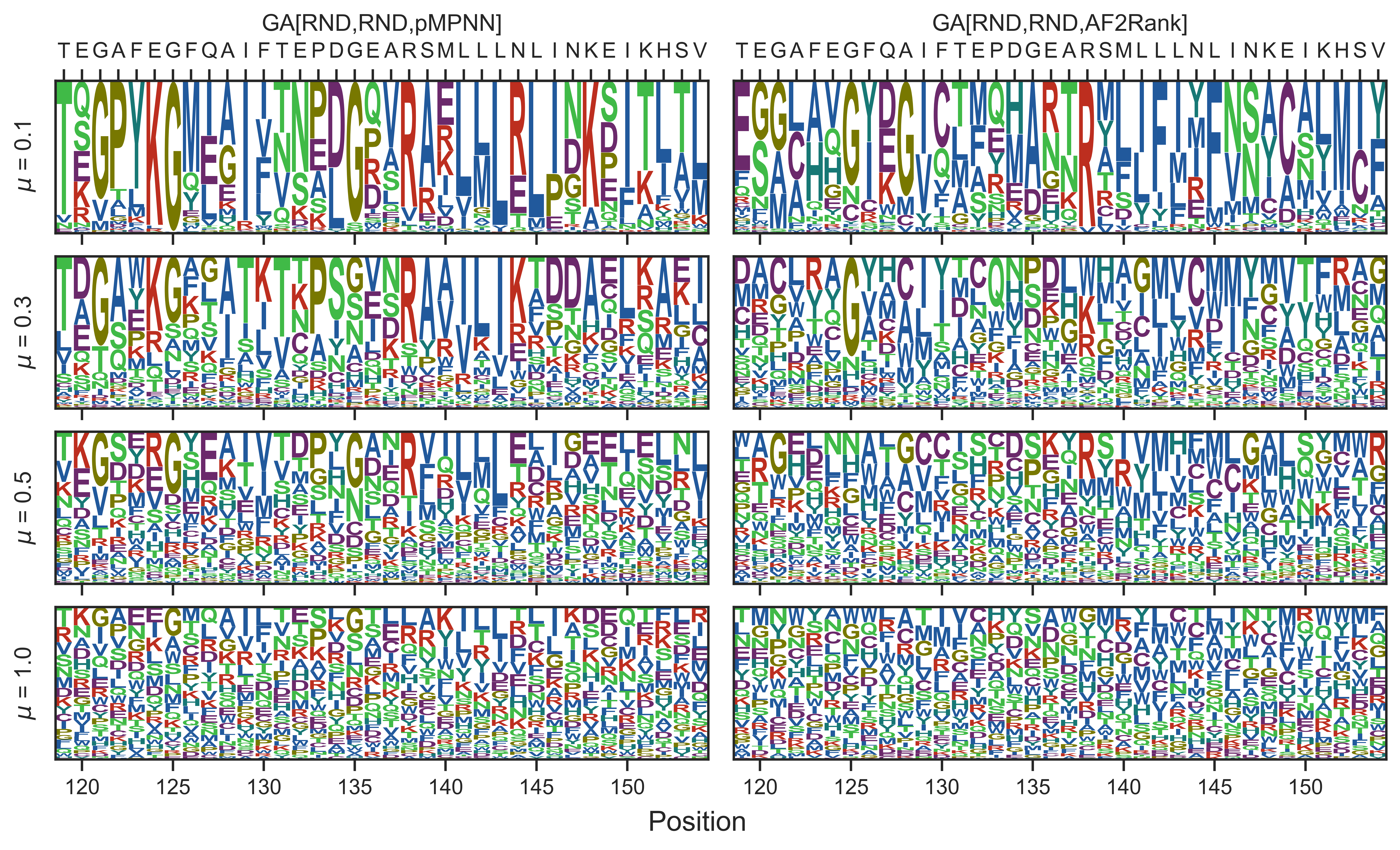

Supplement: S4 Fig — Each panel shows the sequence profiles from the last iteration of the GA[RND,RND,pMPNN] (left column) or the GA[RND,RND,AF2Rank] (right column) setup at a different mutation rate (rows). See also S5 Fig for the cumulative distribution functions of the per-position sequence entropy. (PNG) [file pcbi.1011953.s004.png]

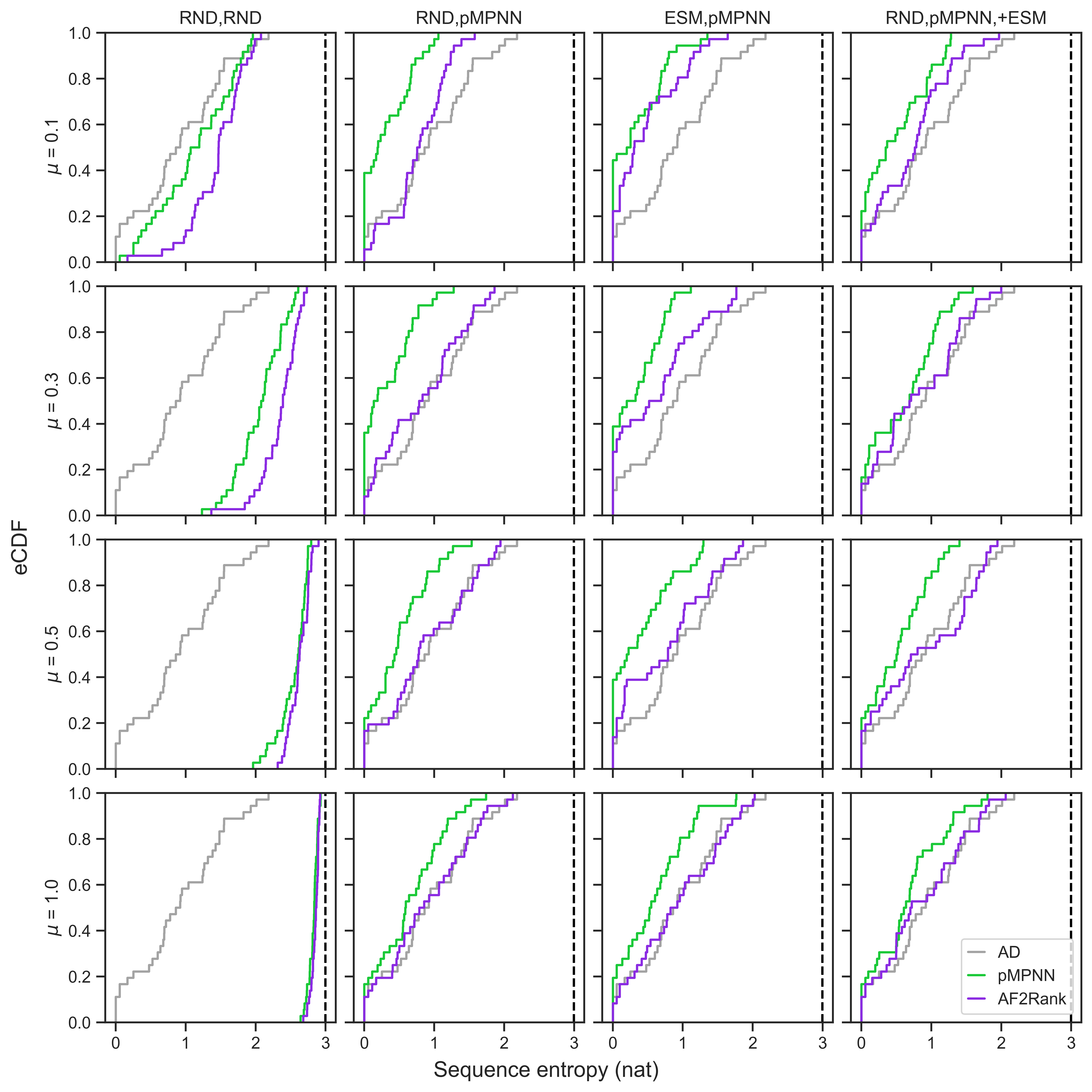

Supplement: S5 Fig — Each panel shows the empirical cumulative distribution function (eCDF) of per-position sequence entropy (base e) for the last iteration population generated with two genetic algorithm setups. Each row represents a mutation rate (μ), and each column represents a mutation operator configuration (“+ESM” indicates that ESM-1v is used as a third objective function); the curves in green indicate that pMPNN-SD log likelihood is used as the objective functions, and the curves in purple indicate that AF2Rank composite score is used as the objective functions. The gray curves are calculated from the pMPNN-AD reference population. The black dashed lines represent ln 20, the entropy of a uniform distribution over the 20 standard amino acid types, which is the theoretical maximum entropy for any discrete distributions that can take on 20 values. See Fig 2 legend for details on the abbreviations used here. (PNG) [file pcbi.1011953.s005.png]

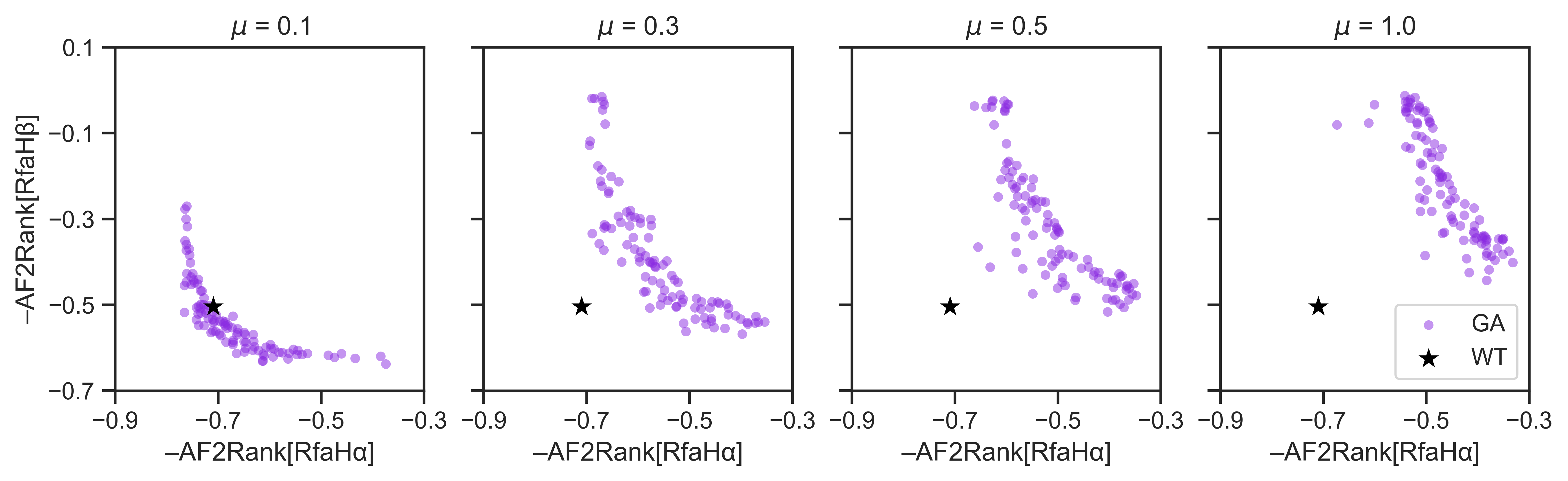

Supplement: S6 Fig — Each panel shows the distribution of the last iteration sequences generated using GA[RND,RND,AF2Rank] at a different mutation rate (purple points), compared to the WT sequence (black star), in the AF2Rank composite score objective space. (PNG) [file pcbi.1011953.s006.png]

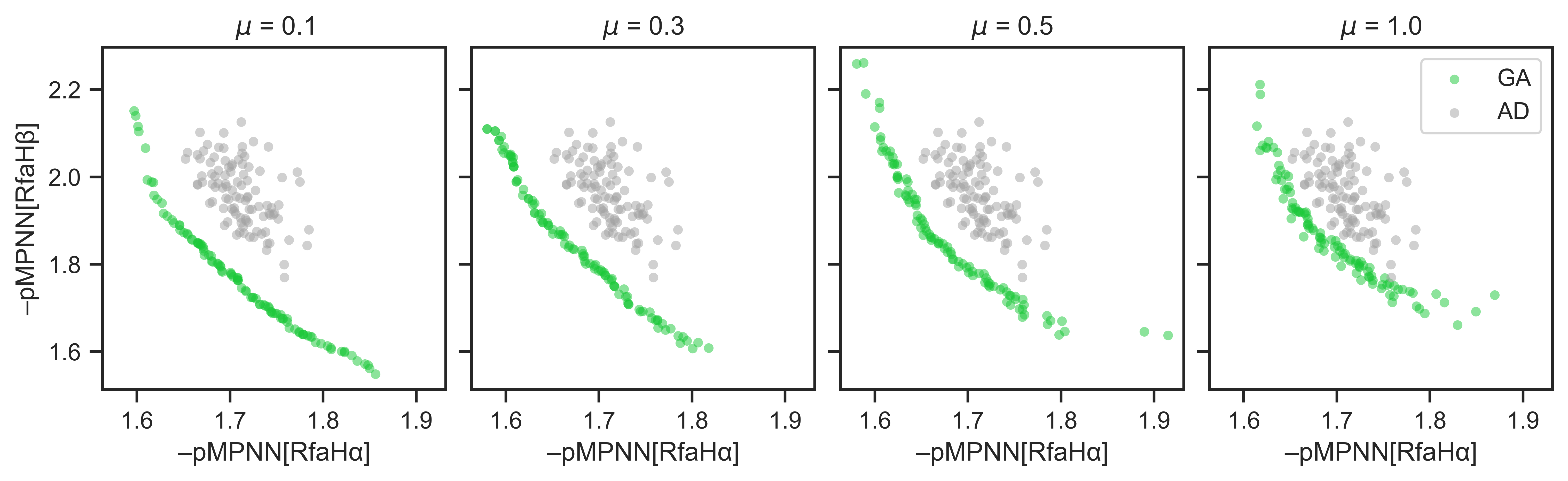

Supplement: S7 Fig — Each panel shows the distribution of the last iteration sequences generated using GA[RND,pMPNN,pMPNN] at a different mutation rate (green), compared to the sequences generated from pMPNN-AD (gray), in the pMPNN-SD log likelihood objective space. (PNG) [file pcbi.1011953.s007.png]

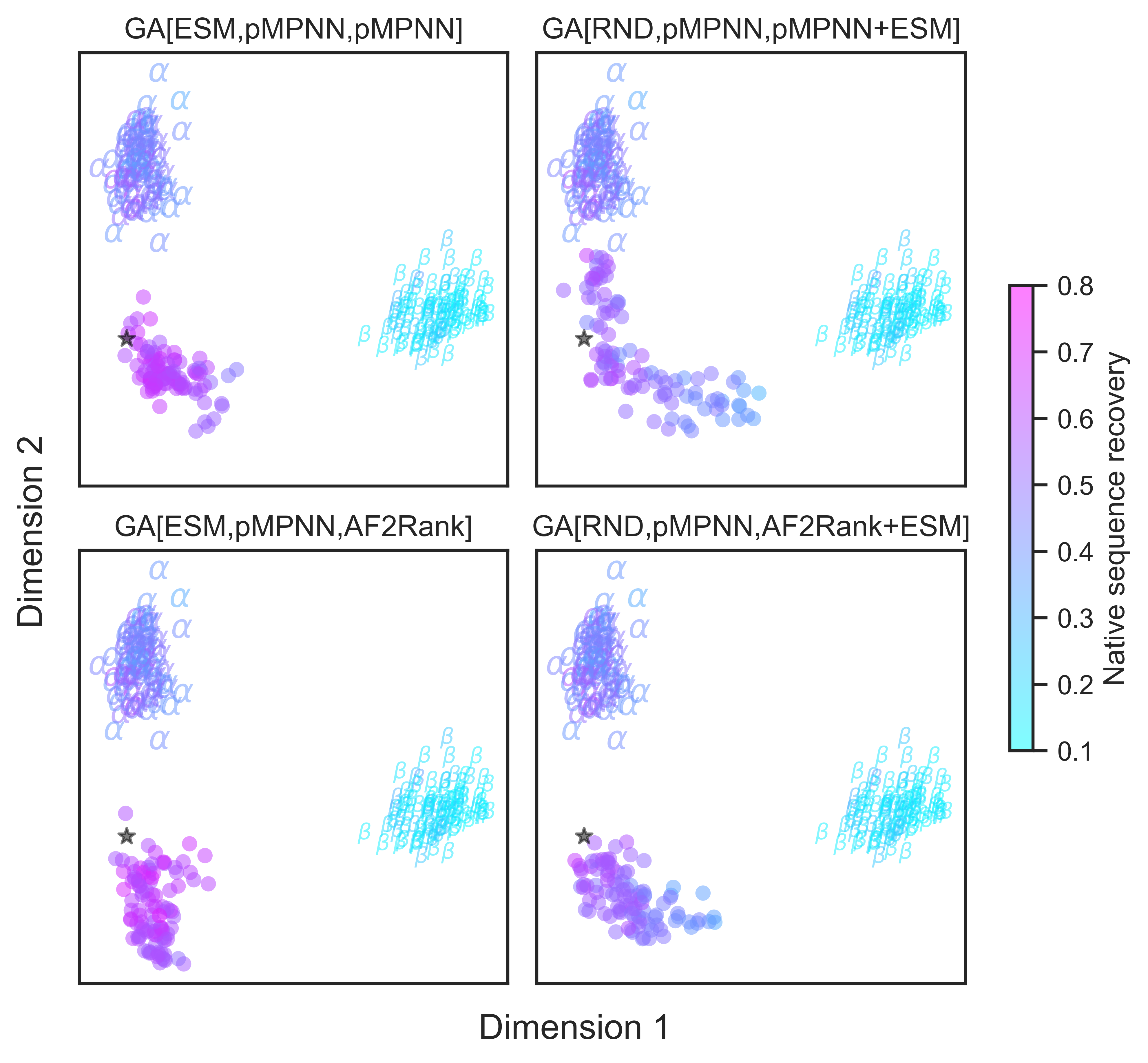

Supplement: S8 Fig — Each panel shows the distribution of the population of the pMPNN-SD sequences and the last iteration population from a GA setup (specified in the title; all with mutation rate 0.3) in a two-dimensional embedding of the sequence space generated using Laplace eigenmaps, as in Fig 3. The sequences are colored by native sequence recovery, except for the WT sequence shown as the black stars; the pMPNN-SD sequences for the RfaHα and RfaHβ states are shown using “α” and “β” as markers. (PNG) [file pcbi.1011953.s008.png]

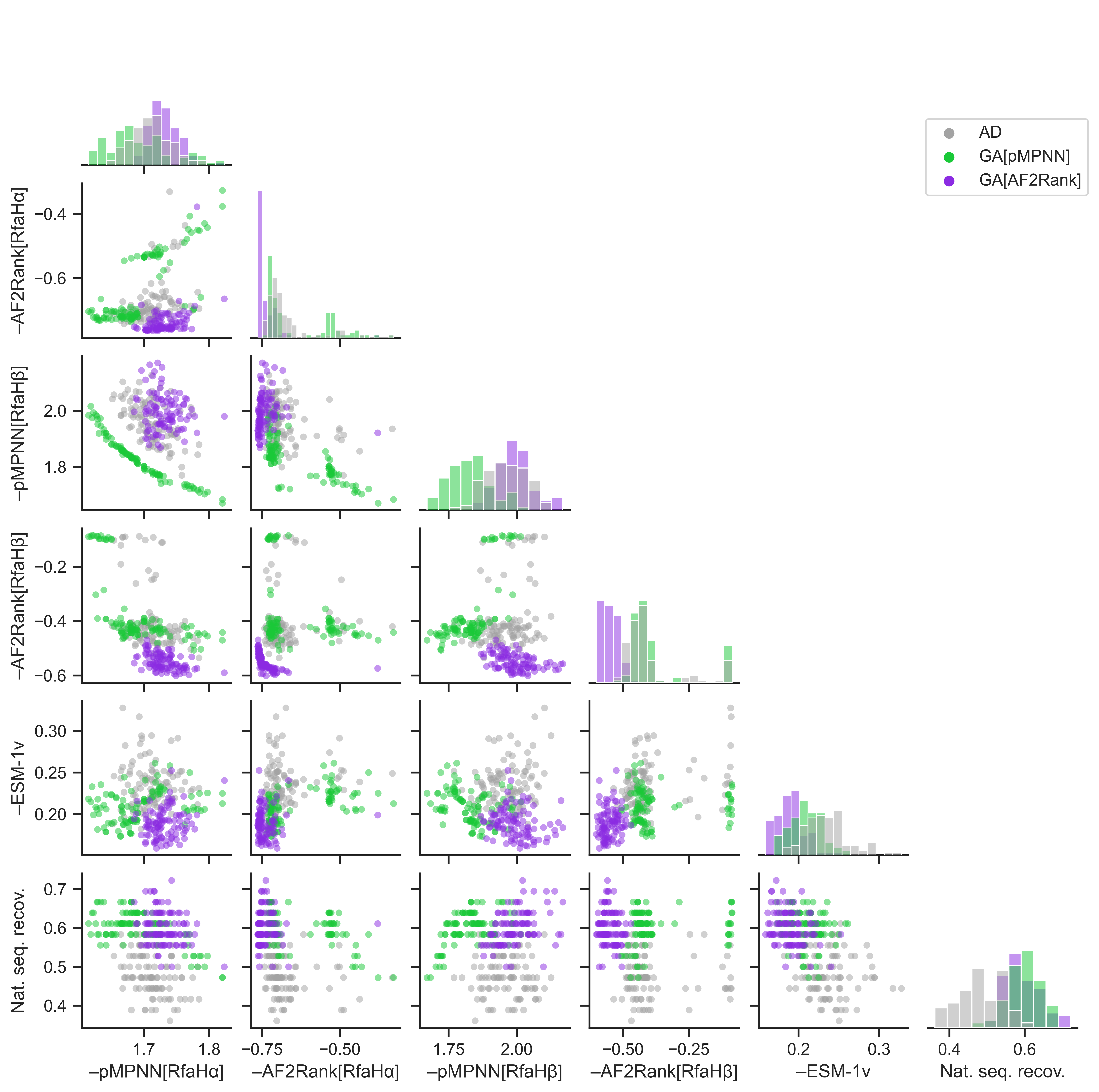

Supplement: S9 Fig — The sequences shown are designed with pMPNN-AD (gray), GA[ESM,pMPNN,pMPNN;μ = 0.3] (green; abbreviated as GA[pMPNN]), and GA[ESM,pMPNN,AF2Rank;μ = 0.3] (purple; abbreviated as GA[AF2Rank]). All GA sequences in this figure refer to the final iteration sequence populations. (PNG) [file pcbi.1011953.s009.png]

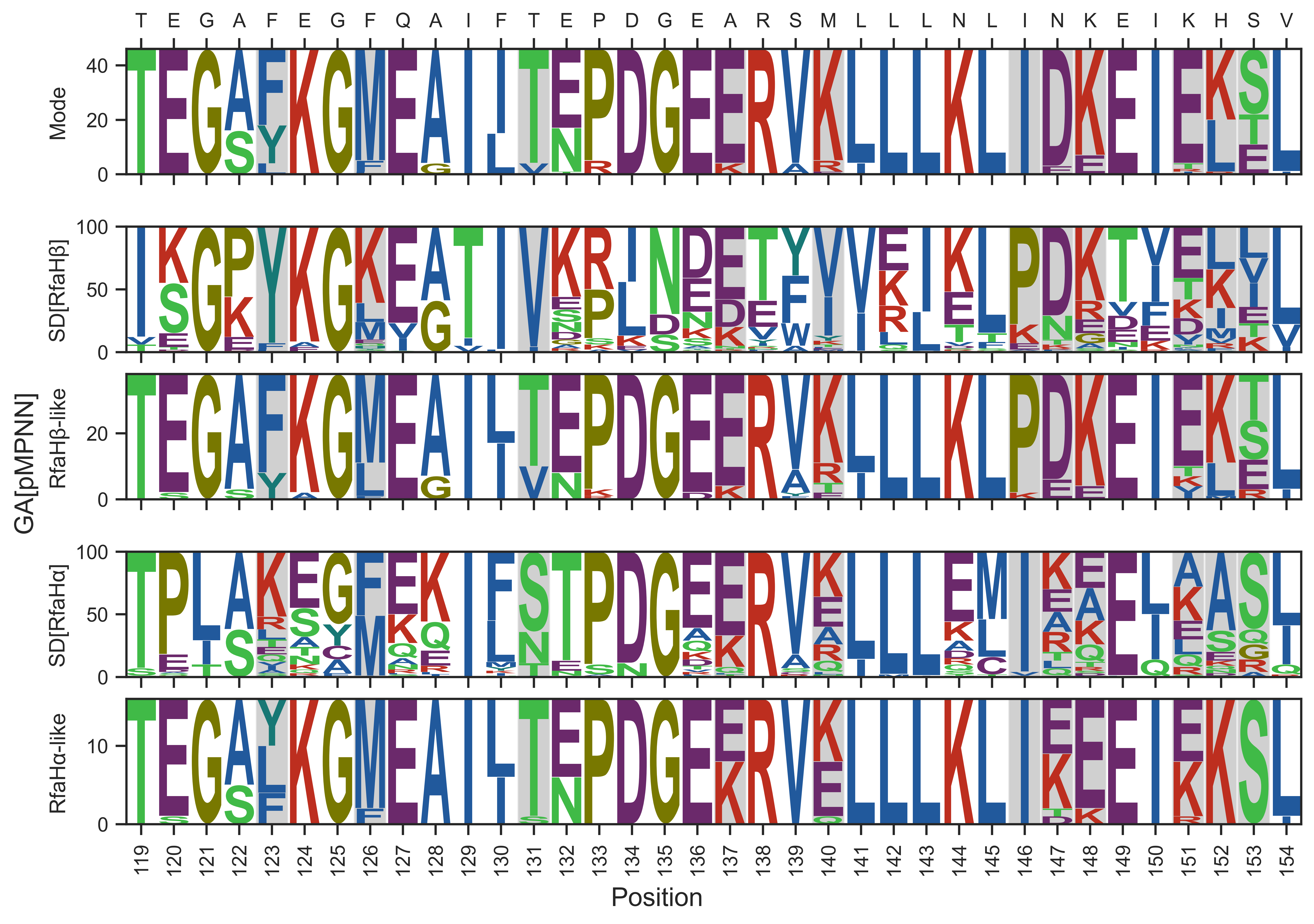

Supplement: S10 Fig — The logo plots for the final iteration sequences designed by GA[ESM,pMPNN,pMPNN;μ = 0.3] (i.e., GA[pMPNN]), which are partitioned here into three groups: “RfaHα-like”, which has bad AF2Rank[RfaHβ] scores (< 0.6); “RfaHβ-like”, which has bad AF2Rank[RfaHα] scores (< 0.3); and “mode”, which consists of the rest of the sequences. See Fig 3A (third panel) for the distribution of these sequences in the AF2Rank objective space. The logo plots for the RfaHα and RfaHβ single-state design (SD) sequences are also shown for comparison. The positions highlighted in gray shading indicate major differences in the recovered residue types among the three subgroups of GA[pMPNN] sequences. (PNG) [file pcbi.1011953.s010.png]

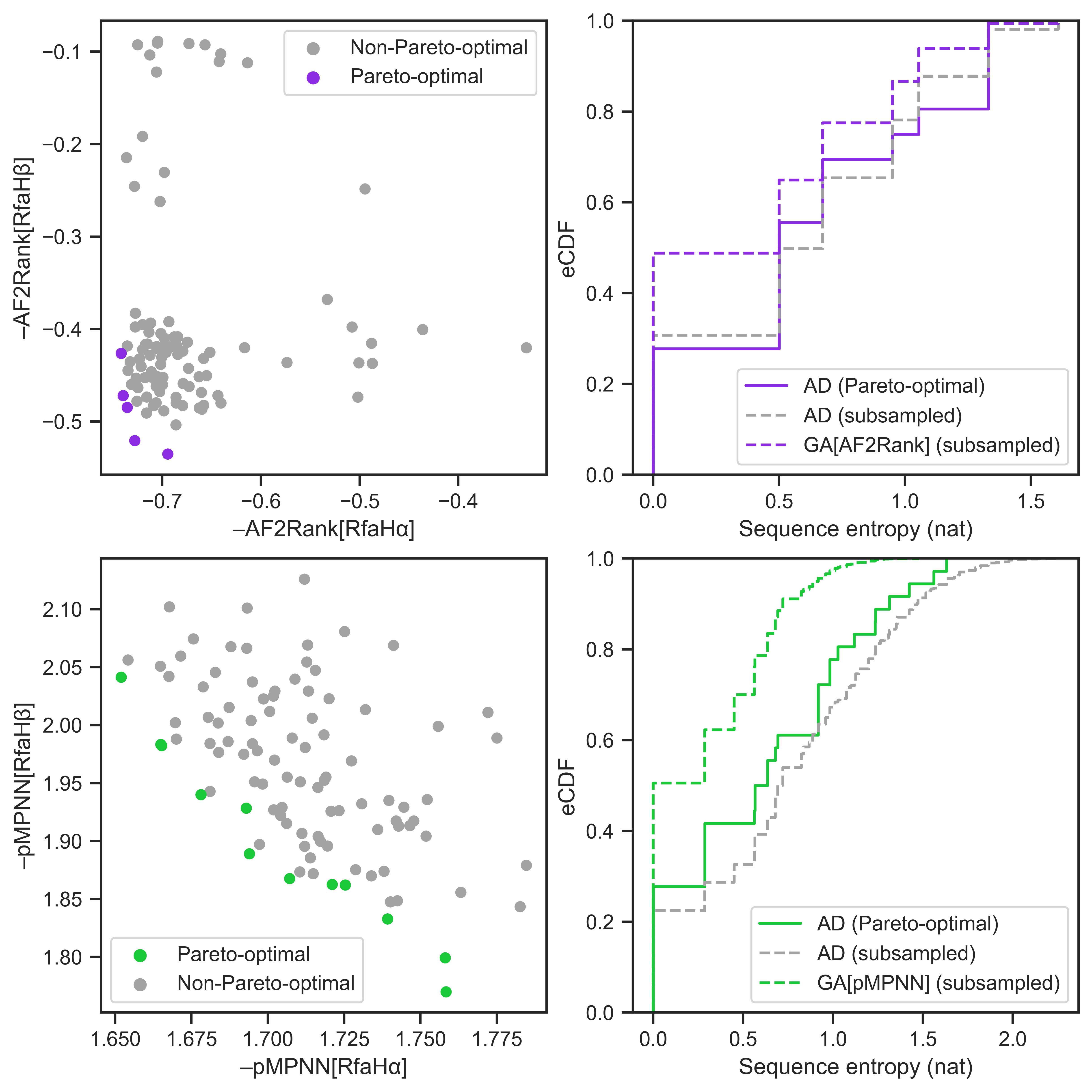

Supplement: S11 Fig — Non-dominated sorting of the 100 pMPNN-AD sequences is performed in the AF2Rank (top row) and pMPNN-SD (bottom row) objective spaces, and the sorting results are shown on the left column. The empirical cumulative distribution functions (eCDF) of the sequence entropies for the sorted, Pareto-optimal solutions are shown on the right column as colored continuous curves. For comparison, sequence entropies are also calculated for a random subsample of the pMPNN-AD sequences of the same size as the Pareto front; this procedure is repeated 100 times and the cumulative distributions of all subsampled sequence entropies is shown as the gray dashed curves. A similar random subsampling method is applied to the GA[AF2Rank] and GA[pMPNN] sequences, and the results are shown as colored dashed curves. (PNG) [file pcbi.1011953.s011.png]

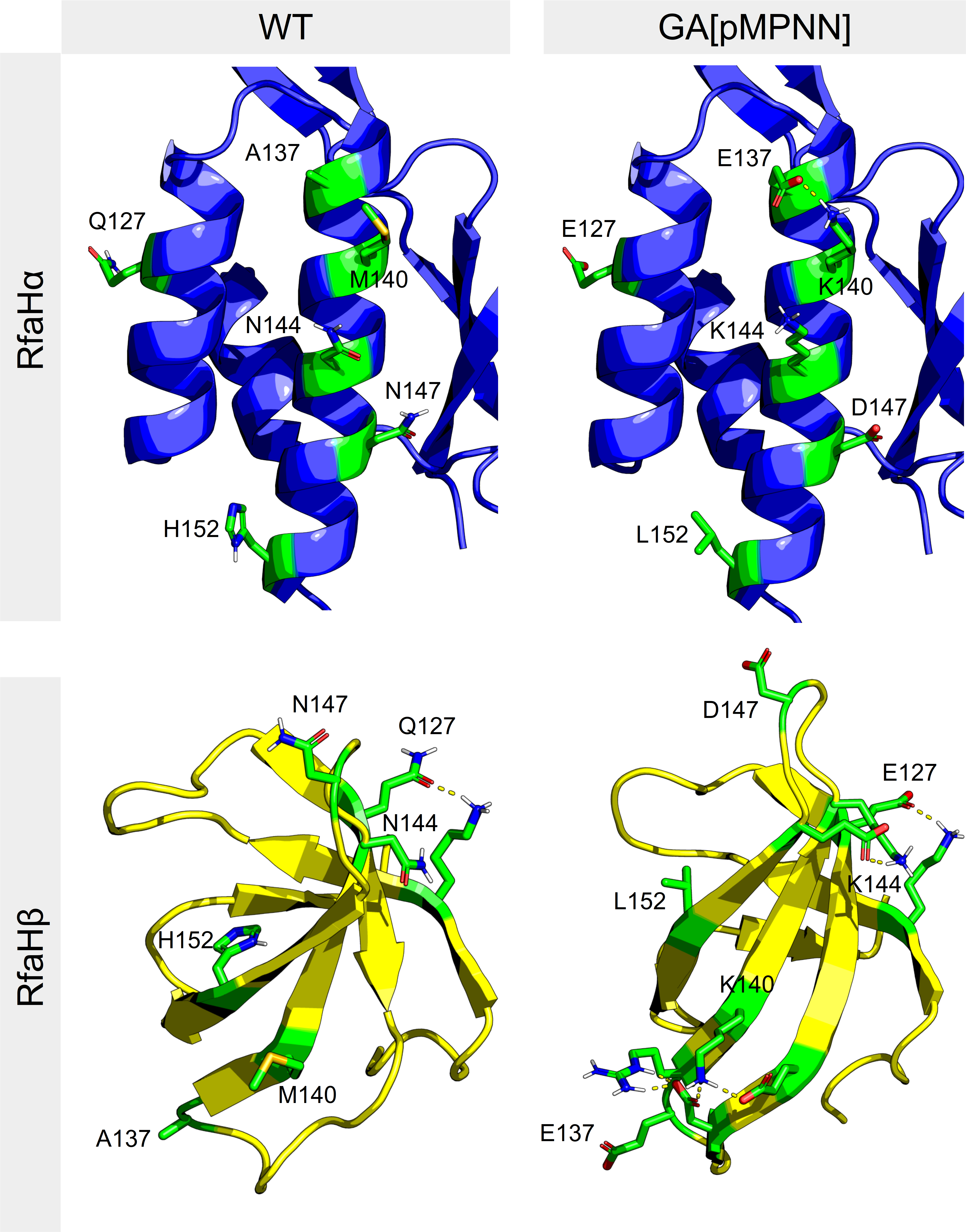

Supplement: S12 Fig — The structure models of the WT (left) and a redesigned (right) sequence chosen from GA[pMPNN] in the RfaHα (top) and RfaHβ (bottom) states are shown in cartoon representation. The sidechain conformations at the positions 127, 137, 140, 147, 152, and any undesigned positions that form salt bridge interactions with these residues, are highlighted in green stick representation; the salt bridge interactions are represented as dashed yellow lines. See Methods for how the redesigned sequence is chosen and how its structural model is generated. (PNG) [file pcbi.1011953.s012.png]

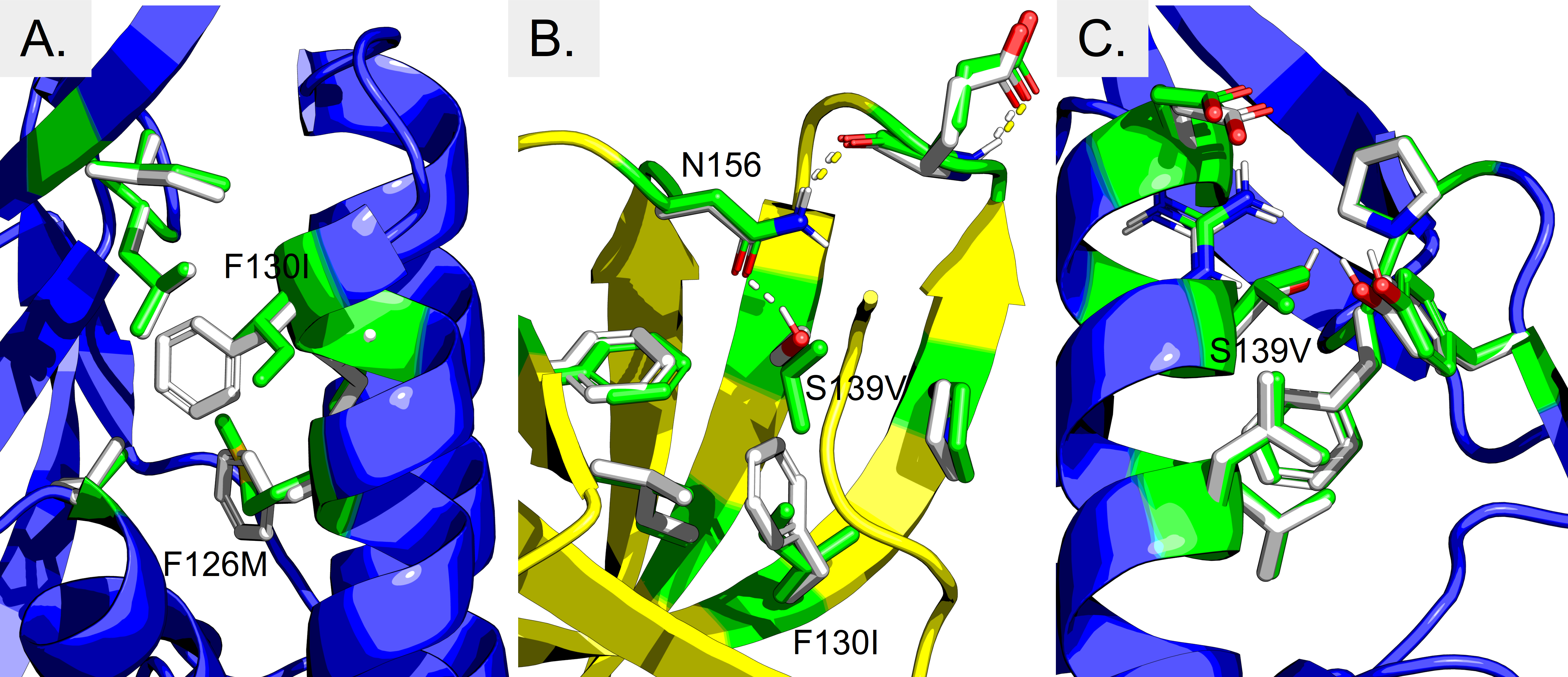

Supplement: S13 Fig — The local structural environment around F130I in the RfaHα state is shown in A, and the local environment around S139V in the RfaHβ and RfaHα states are shown in B and C, respectively. The backbone of the redesigned structure is shown in cartoon representation, while the sidechains of the WT and redesigned residues are shown in white and green stick representation, respectively. The hydrogen bonding interactions are shown with dashed lines, with those in the WT structure in white and those in the redesigned structure in yellow. The redesigned sequence and its structural models shown here are the same as that in S12 Fig. (PNG) [file pcbi.1011953.s013.png]

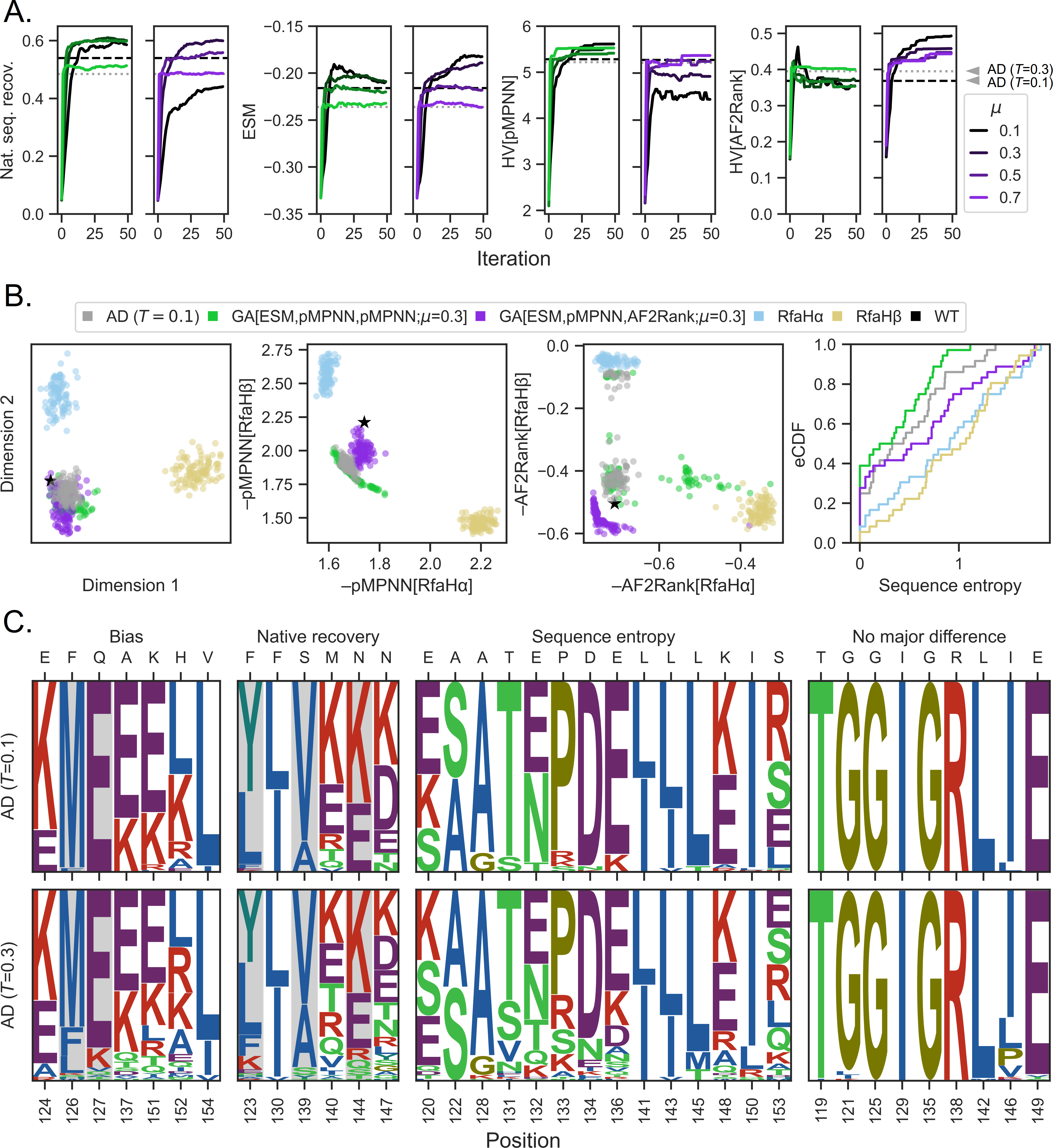

Supplement: S14 Fig — A. Progression of GA[ESM,pMPNN,pMPNN] and GA[ESM,pMPNN,AF2Rank] simulations, reproduced from Fig 2. The dashed black lines represent the population averages from the pMPNN-AD sequences at T = 0.1, while the dotted gray line represents averages at T = 0.3, as per Fig 2. B. Distribution of pMPNN-SD, pMPNN-AD, and GA sequences, reproduced from Fig 3A, but with the pMPNN-AD population from the T = 0.1 simulation. C. Logo plots for sequences generated with pMPNN-AD at T = 0.1 (top) and T = 0.3 (bottom). The residue positions highlighted in gray shading show reduced or no native sequence recovery at T = 0.1 compared to T = 0.3. (PNG) [file pcbi.1011953.s014.png]

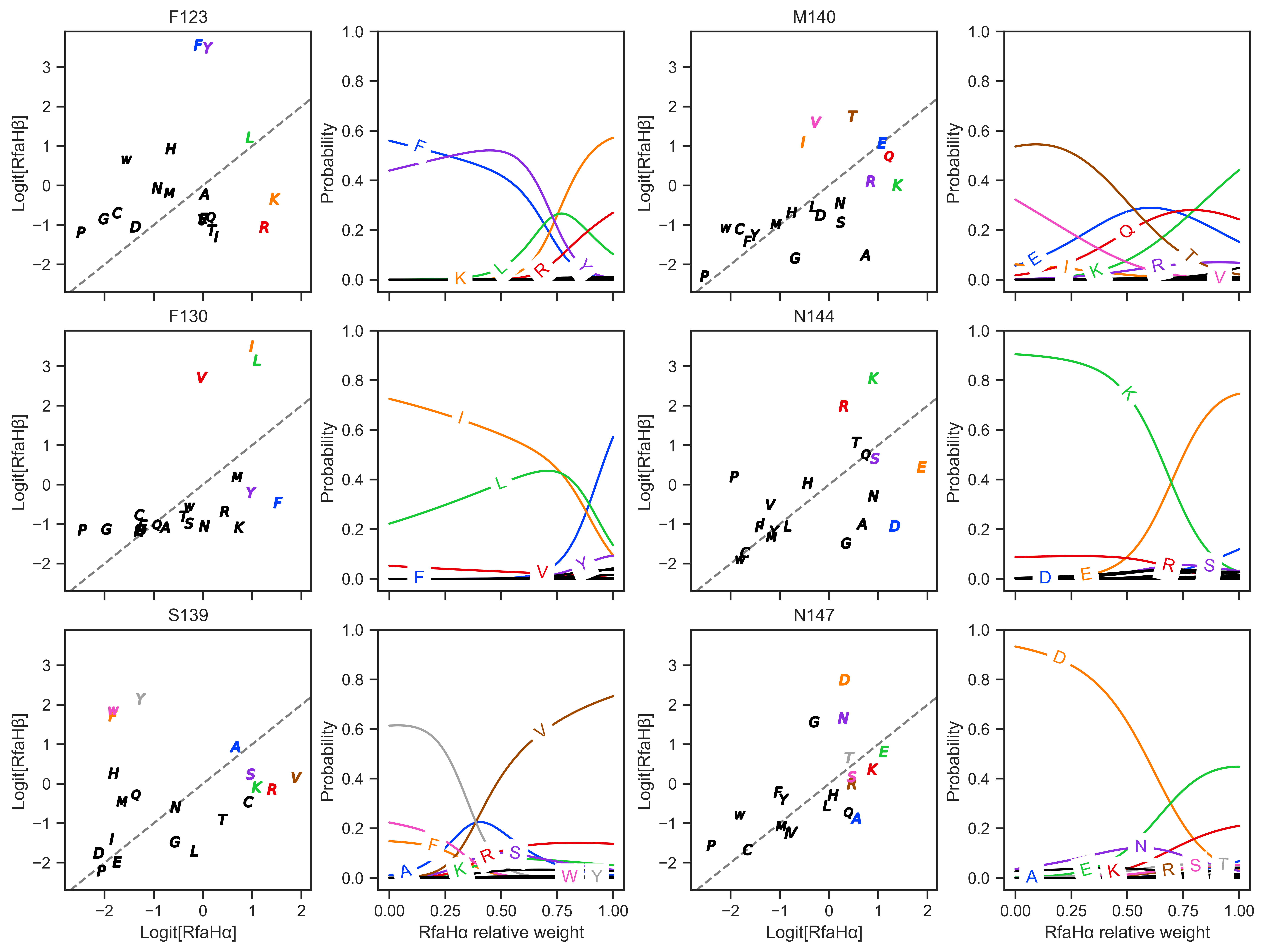

Supplement: S15 Fig — For F123, F130, S139, M140, N144, and N147 (i.e., positions identified in Fig 3C in the “native recovery” category), we perform a single-position sequence design simulation with pMPNN-AD to extract the raw logits (not scaled by temperature or normalized by softmax) of all 20 standard residue types for the two RfaH states (first and third columns). The dashed gray lines represent the y = x diagonal lines. Then, we ask how the probability assigned to each residue type (at temperature 0.3) changes as a function of the relative weight for the RfaHα state (second and fourth columns). If a residue type is ever assigned a probability > 0.05 as a function of state weight at a position, then the residue is shown in color on the panels associated with the position; otherwise the residue is shown in black. Note that the relative proportions of decoded residues using pMPNN-AD shown in Fig 3 will not necessarily match those at relative weight 0.5 shown here, because in practice sequence decoding at any given position is usually not performed with the full WT sequence context. (PNG) [file pcbi.1011953.s015.png]

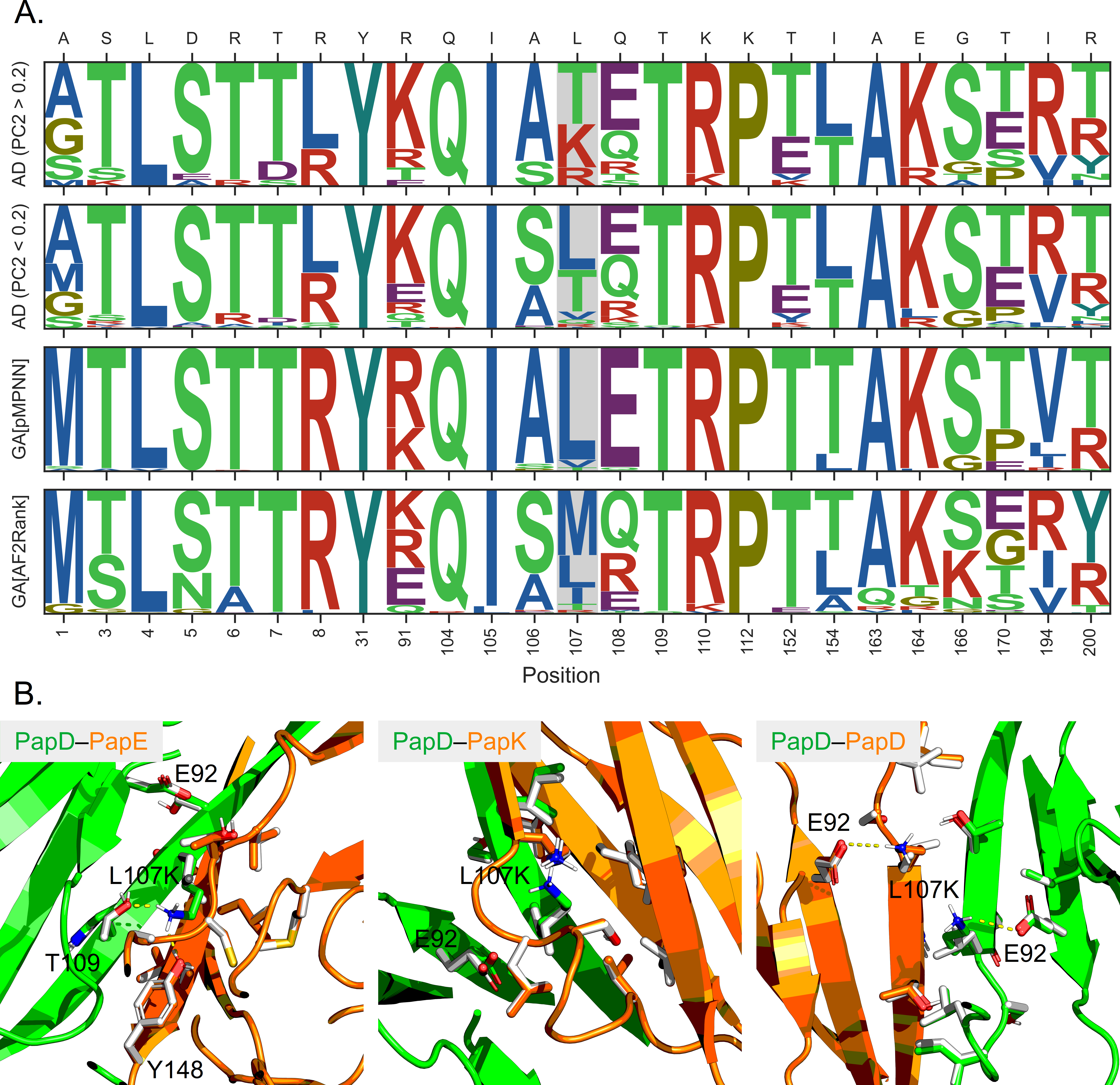

Supplement: S16 Fig — A. Logo plots for the sequences designed using pMPNN-AD (abbreviated as AD) (top two rows), the last iteration of GA[ESM,pMPNN,pMPNN;μ = 0.3] (abbreviated as GA[pMPNN]) (third row) and the last iteration of GA[ESM,pMPNN,AF2Rank;μ = 0.3] (abbreviated as GA[AF2Rank] (last row). For pMPNN-AD, the redesigned sequences are split into two subpopulations, depending on the position of the sequences in the AF2Rank composite score objective space (Fig 5B, third panel): sequences that reside in the upper half of the principal component space (defined as having a second principal component (PC2) value greater than 0.2) are shown in the top row, and sequences that reside in the lower half are shown in the second row. The residue distributions at L107 are highlighted in gray. B. Structural analysis of L107K. A sequence from pMPNN-AD containing the L107K mutation is selected and used to generate the structural models shown here (see Methods). In each panel, PapD is shown in green cartoon representation, and its binding partner is colored in orange; the binding partners are, from left to right: PapE, PapK, and PapD. All residue sidechains containing atoms within 4 Å of the L107K sidechain are represented in stick models, and hydrogen bonds within the neighborhood are represented by dotted yellow lines; the sidechains from the WT structures are colored in white, while those from the redesigned structural model are colored in the same way as the backbone cartoon representation. In each panel, E92 is labeled, and residues that form polar and charged interactions with L107K are also labeled. In particular, while E92 forms salt bridge interactions with L107K at the PapD–PapD interface, the geometry at the PapD–PapE and PapD–PapK interfaces do not appear to allow for direct salt bridge interactions between E92 and L107K. (PNG) [file pcbi.1011953.s016.png]

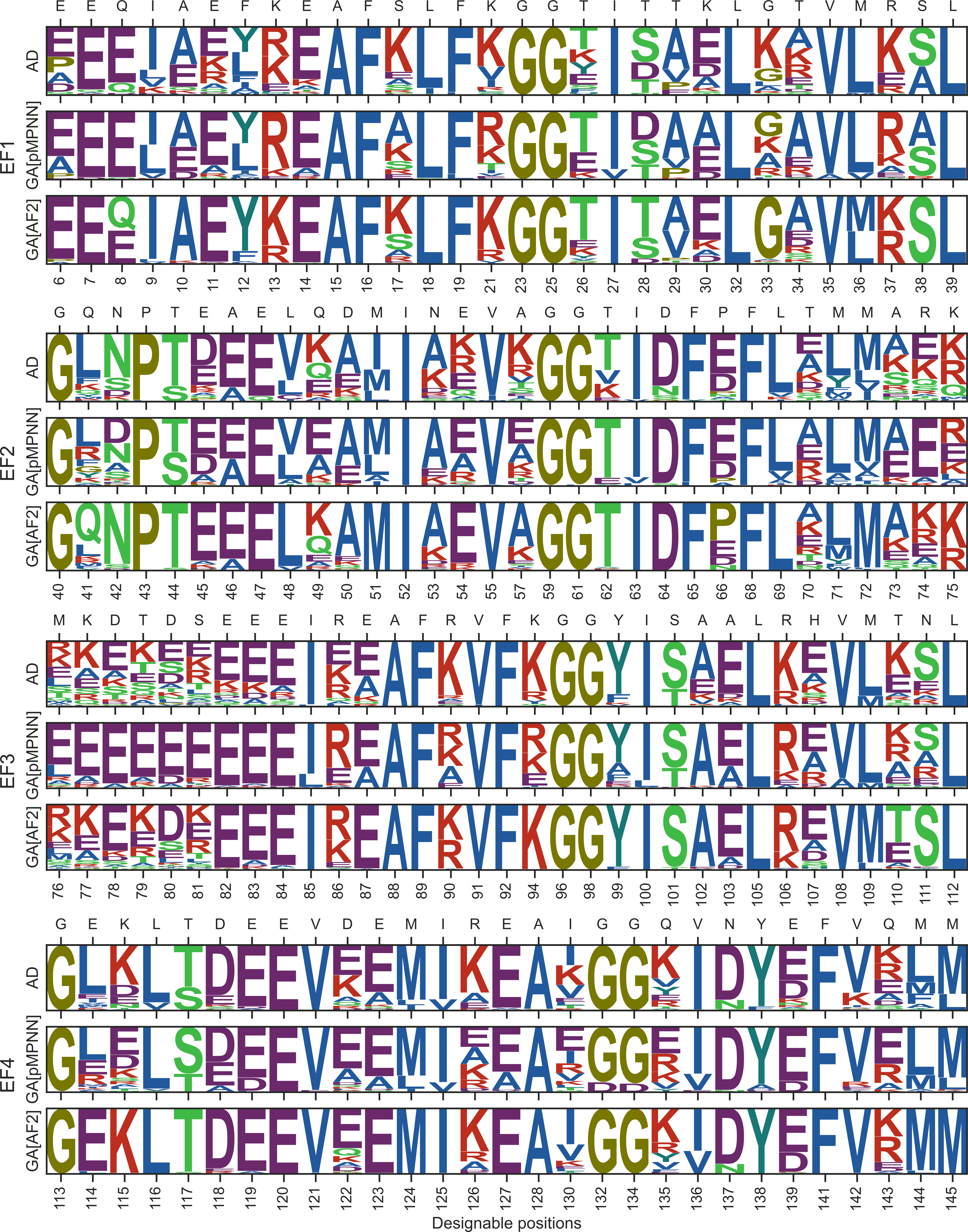

Supplement: S17 Fig — Logo plot for redesigned CaM sequences from pMPNN-AD (abbreviated as AD), the last iteration of GA[ESM,pMPNN,pMPNN;μ = 0.3] (abbreviated as GA[pMPNN]) and the last iteration of GA[ESM,pMPNN,AF2Rank;μ = 0.3] (abbreviated as GA[AF2]). Due to the length of the designable sequence, the logo plot is split into four residue blocks, roughly corresponding to the four EF hands of CaM: EF1 (residues 6–39), EF2 (residues 40–75), EF3 (residues 76–112), and EF4 (residues 113–145); residues within the 6–145 range that are not redesigned (remain wild-type) are not depicted in the Figure (see Methods). (PNG) [file pcbi.1011953.s017.png]

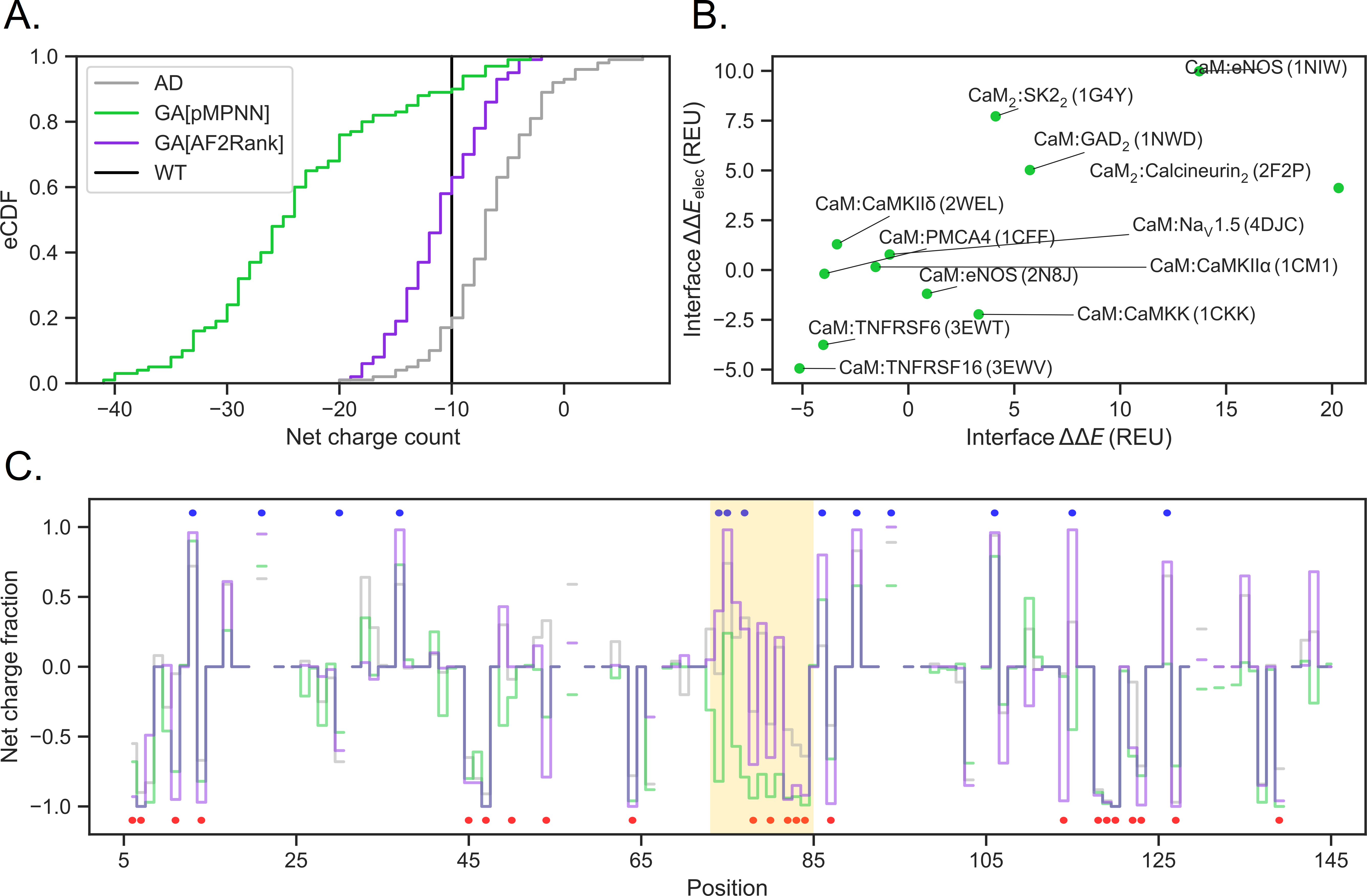

Supplement: S18 Fig — A. Empirical cumulative distribution functions (eCDF) of net charge count for sequences designed using pMPNN-AD (abbreviated as AD), the last iteration of GA[ESM,pMPNN,pMPNN;μ = 0.3] (abbreviated as GA[pMPNN]) and the last iteration of GA[ESM,pMPNN,AF2Rank;μ = 0.3] (abbreviated as GA[AF2Rank]); the WT net charge count is shown as the black vertical line. The net charge count is defined as the number of K and R, minus the number of D and E, in the designable positions. B. Interface energetics for a representative sequence designed by GA[pMPNN]. See Methods on how the sequence is chosen and details on the calculation of interface energetics. The net charge count of this chosen sequence is –29, and only CaM states with a binding partner are included in this analysis. REU stands for Rosetta energy unit. C. Distributions of charged residues in the sequences redesigned by pMPNN-AD, GA[pMPNN], and GA[AF2Rank] (same sequences and color scheme as panel A). The net charge fraction at each position is defined as the net charge count at that position, summed over the design population, and divided by the size of the design population. Positions with a K or R as the WT residues are represented by a blue dot, and positions with a D or E as the WT residues are represented by a red dot. The central portion of the linker region (residues 74–84) enriched with charged residues is highlighted in light yellow. The residues not redesigned are represented as gaps in the traces. (PNG) [file pcbi.1011953.s018.png]

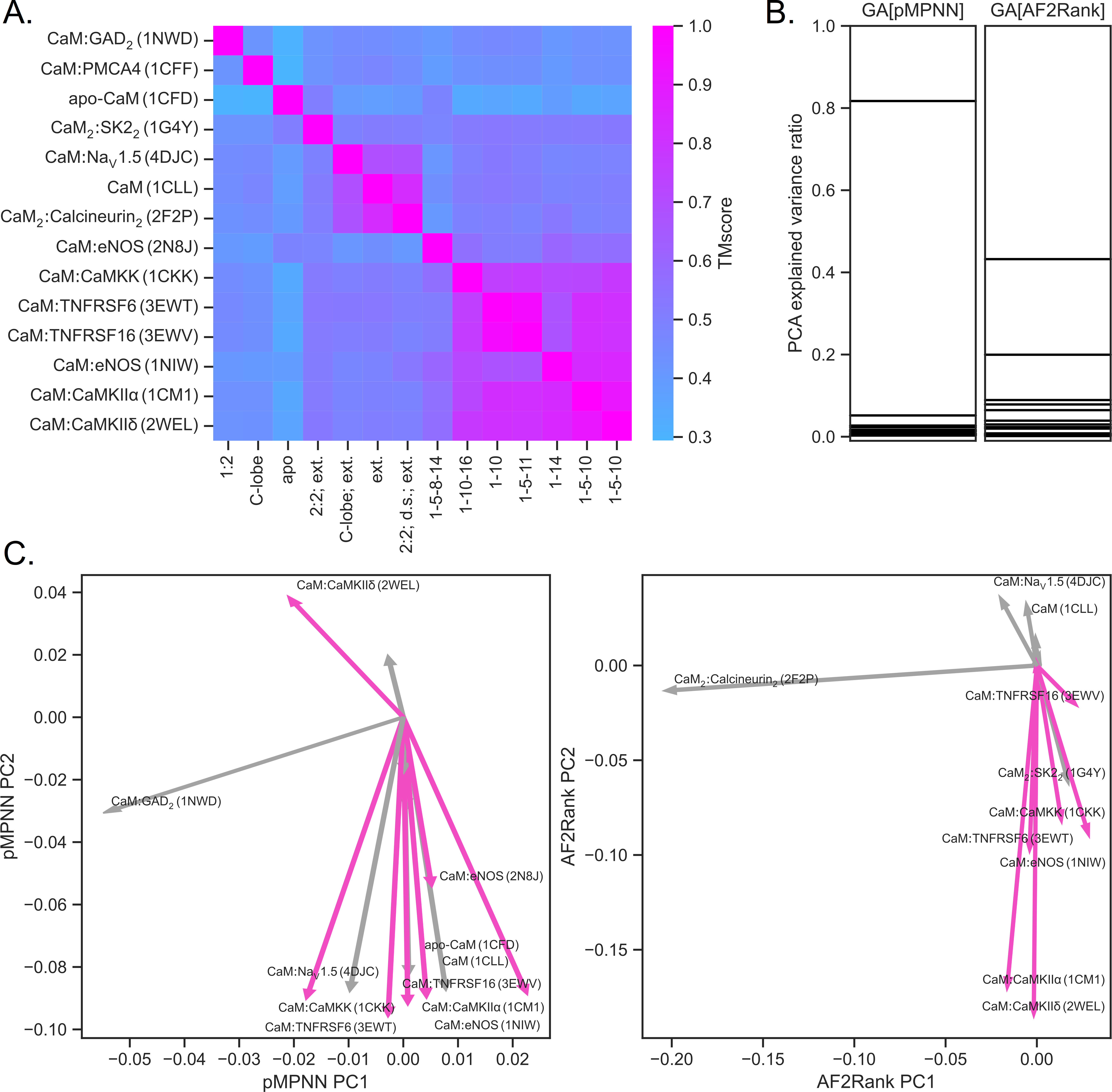

Supplement: S19 Fig — A. Pairwise TM-scores for the CaM structures used for design. The TM-scores are calculated using residues 6–145 of CaM only. The pairwise TM-score matrix is arranged using a single-linkage hierarchical clustering to highlight the correlations within the set of CaM structures. Each CaM structure is described by two labels: the complex represented by the structure, followed by the PDB ID (column), and the binding mode represented by the structure (row). Unless labeled as “apo”, the structures used were resolved in the presence of Ca2+. For the binding mode labels: “1:2” and “2:2” indicates a 1:2 and 2:2 stoichiometric ratio between CaM and the binding partner, respectively; “C-lobe” indicates that the binding partner is only associated with the C-terminal lobe of CaM; “ext.” indicates that CaM is in an extended conformation; “d.s.” indicates a domain-swapped CaM dimer; a label with numbers connected by dashes indicates that the structure is described by a canonical binding mode (the numbers represent the primary sequence distances of the anchoring hydrophobic residues on the binding partner). B. The eigenvalue spectra from principal component analysis (PCA) of the GA[ESM,pMPNN,pMPNN;μ = 0.3] (abbreviated as GA[pMPNN]) and GA[ESM,pMPNN,AF2Rank;μ = 0.3] (abbreviated as GA[AF2Rank]) simulations. The GA[pMPNN] PCA analysis is done over the last iteration sequences generated by GA[pMPNN], in the pMPNN-SD log likelihood score objective space, while the GA[AF2Rank] PCA analysis is done over the last iteration sequences generated by GA[AF2Rank], in the AF2Rank composite score objective space. The two outlier sequences in Fig 5D (second panel) are removed prior to the PCA analysis. For each PCA, the eigenvalues are divided by the sum of all eigenvalues to produce the explained variance ratio (i.e., fraction of the total variance in the objective space explained by each corresponding principal component). Note that the PCAs examined here are different from those used for visual [file pcbi.1011953.s019.png]

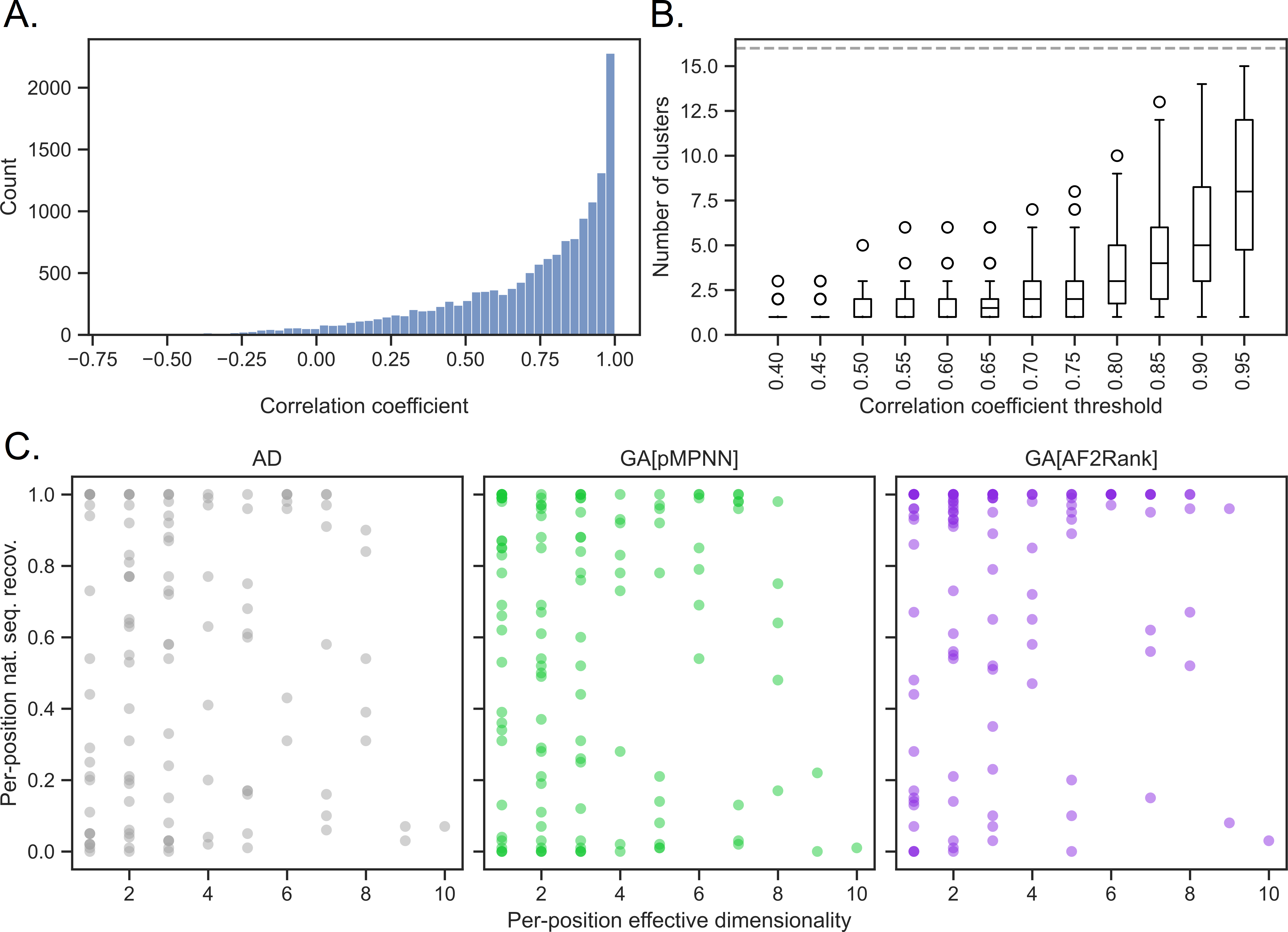

Supplement: S20 Fig — A. The distribution of pairwise correlation coefficients for pMPNN-SD logits. The per-position pMPNN logit vectors are extracted as for RfaH (see S15 Fig). For each designable position, the Pearson’s correlation coefficient is calculated for each pair of logit vectors (i.e., for each pair of CaM chains), and the correlation coefficients for all positions are pooled to generate the histogram. B. The distribution of cluster numbers as a function of correlation coefficient threshold. For each designable position, a single-linkage hierarchical clustering is performed on the Pearson’s correlation coefficient matrix over the pMPNN logit vectors. The logit vectors are then clustered by cutting the resulting dendrogram at a given correlation coefficient threshold. The number of clusters per position, which we interpret as the per-position effective dimension, is pooled to generate the box plot. The box plot extends from the first to the third quartile of the distributions, with the median line shown in between; the fliers represent outliers of the distributions. The dashed line at 16 indicates the maximum possible dimensionality; note that this is higher than the 14 states used to define the design problem, because two of the states contain CaM dimers. C. Correlation between the per-position effective dimensionality (i.e., the number of clusters per position in panel B) and per-position native sequence recovery (nat. seq. recov.) From left to right, each panel represents the correlation for sequences designed using pMPNN-AD (abbreviated as AD), the last iteration of GA[ESM,pMPNN,pMPNN;μ = 0.3] (abbreviated as GA[pMPNN]) and the last iteration of GA[ESM,pMPNN,AF2Rank;μ = 0.3] (abbreviated as GA[AF2Rank]). The per-position native sequence recovery is computed as the number of designed sequences with the WT residue at a given position, divided by the sequence population size. The per-position effective dimensionality is computed using a correlation coefficient threshold of 0.8 [file pcbi.1011953.s020.png]

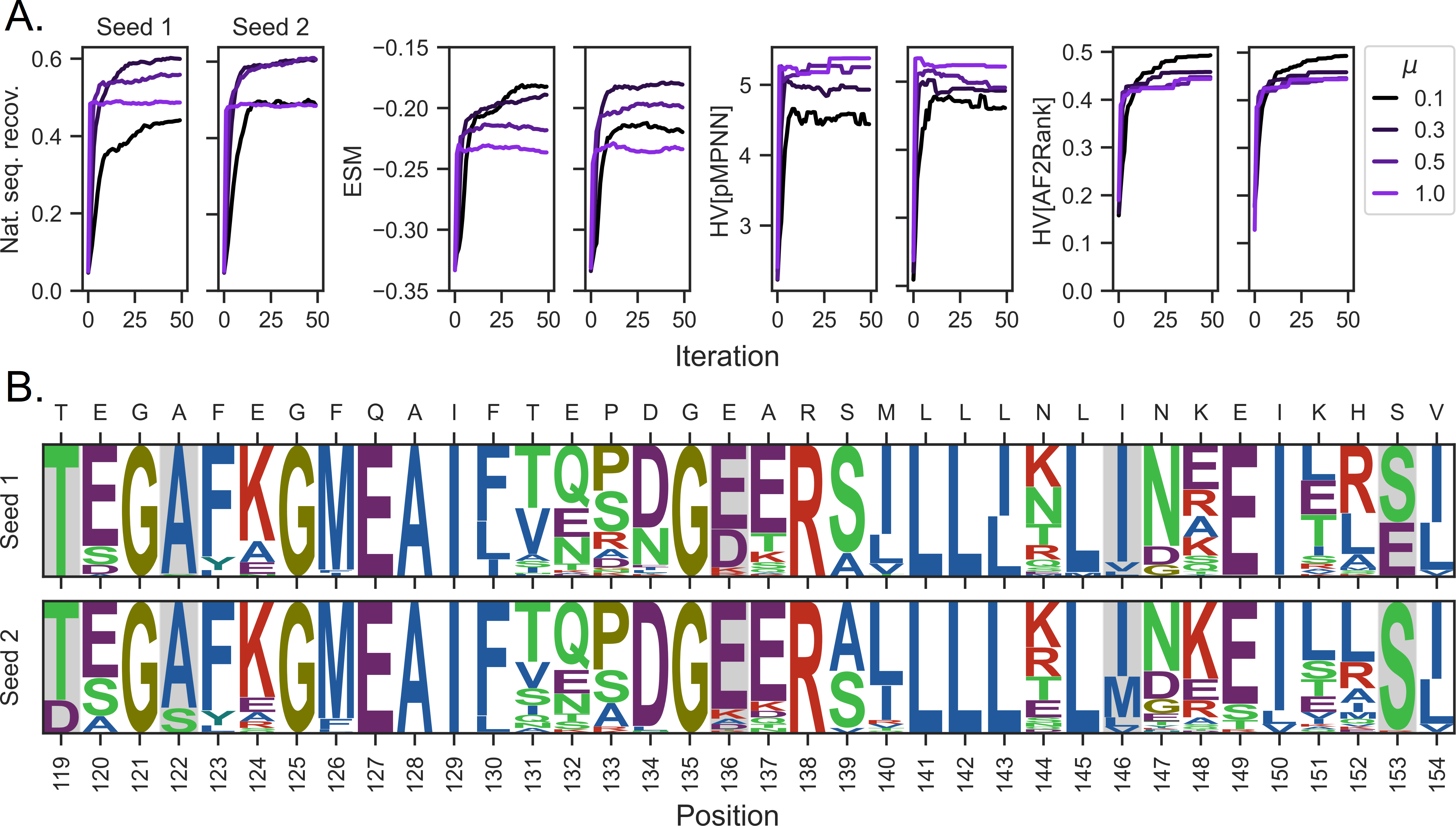

Supplement: S21 Fig — A. Comparison of convergence metrics for GA[ESM,pMPNN,AF2Rank] simulations at four mutation rates using two different starting random seeds. See Fig 2 for more details on the metrics. The simulation results presented in the rest of this work is based on seed 1. B. Comparison of the last iteration sequence logos at the mutation rate μ = 0.3 for the two random seeds. Positions with major differences in the recovered sequence profiles are highlighted in gray shading; in both simulations, the WT residue types are recovered at these positions, but the simulations differ in terms of the alternative residue types recovered at these positions. (PNG) [file pcbi.1011953.s021.png]
