# Supplementary material for: An integrative approach to protein sequence design through multiobjective optimization
Source: PLoS Comput Biol. 2024 Jul 11;20(7):e1011953. doi: 10.1371/journal.pcbi.1011953 (PMC11265717; doi:10.1371/journal.pcbi.1011953)
Supplement: Attachment — Submitted filename: multistate_design_response_letter.pdf [file pcbi.1011953.s022.pdf]

Dear Editor:

Please find below our point-by-point response to the reviewers' comments. The reviewers' original comments are in black, our responses are in blue, and changes made to the manuscript in response to the reviewers' comments are highlighted in yellow.

Reviewer #1:

This manuscript describes methodological advances relevant to protein design. Specifically, it examines multi-objective optimization using recently introduced machine-learning approaches for sampling sequence space and evaluating models within a genetic algorithm framework. The application is a fold-switching protein that presents significant challenges for design via established protocols.

Overall, the analysis is interesting, but a main concern is that the methodology is tested only for one system, and it is unclear whether the findings have broader relevance for other systems. It seems that at least several different systems should be tested before conclusions with any level of significance can be obtained.

To assess whether the results from RfaH can be generalized to other systems, we performed and analyzed additional benchmark simulations on two more model systems: PapD and CaM. These two model systems were chosen for two reasons. First, they represent common challenges in multistate design outside of foldswitching proteins: PapD involves a multi-specific binding interface, and CaM involves both a multi-specific binding interface and changes to secondary and tertiary structure. Second, these model systems need to be represented in higher-dimensional objective spaces (3 for PapD and 14 for CaM), thus providing a good test of whether the genetic algorithm setups can be applied to higher-dimensional settings, a known challenge for this class of algorithms.

Our analysis suggests that for these two new model systems, the genetic algorithm setups can still outperform a direct application of ProteinMPNN by reducing the bias and variance in native sequence recovery, similar to the conclusions drawn for RfaH. Please see the three new subsections in the Results section (pages 22–29) for a detailed account of our analysis results. The Methods section is updated accordingly to reflect the two new model systems; we have also made various minor changes in the text to clarify which model system is being referred to.

In terms of presentation, the paper is dense at many places and lacking clarity. Especially at the beginning it is difficult to understand what exactly was done. It would help to have a flowchart in addition to or instead of Figure 1 that clearly illustrates the protocol, especially with respect to what type of output (sequence, structure, score) is obtained based on what type of input with the different tools and how this data is used within the genetic algorithm optimization framework. It probably would also help to explicitly state in the introduction what the main benchmark goal is

(native sequence recovery based on a given structure, or in this case a set of structures) as that may not be obvious to readers that are not intimately familiar with protein design method assessment.

We have now included a new, detailed flowchart (see the new S1 Fig) that illustrates the protocol, and added additional clarifications in the introduction.

Additional minor comments/questions:

Why were residues 97-107 deleted from the 2LCL structure and does it matter that the Hbeta and Halpha structures apparently have different amino acid lengths?

We added additional discussions of this issue at the end of the subsection “Structure preparation” in the Methods section. To summarize briefly here: In 2LCL, residues 1–96 are not resolved and residues up to 107 are highly disordered, so we omitted them from our structure-dependent design approach. In addition, having the same chain lengths is not a requirement for tied residue design in ProteinMPNN, which only requires the tied, designable residues to be present in all relevant states. However, the fact that some chains have different lengths do have two consequences. First, the local environment around the undesigned missing residues may be different from that of the full-length protein, which may add a constant bias to all simulation setups; but because the bias is constant in all setups, it does not affect our comparative analysis of the setups. Second, structure-based metrics may not be comparable across states due to chain length differences. As such, these metrics should only be compared within each state, not between them; this was indeed how these metrics were used in the genetic algorithms and interpreted in this work.

How sensitive is the protocol to the initial structural ensemble?

We are not sure what the “initial structural ensemble” refers to in this question. In the genetic algorithm setups, there is only a single input structure per state (generated by Roestta structural relaxation, see Methods) and these input structures are not modified during simulations.

If the reviewer was referring to the initial sequence population, please see S21 Fig (previously S14 Fig), where we examined the effect of different starting random seeds on the genetic algorithm simulation outcomes; an important function of the starting random seed is that it controls how the WT sequence is randomized to generate the initial sequence population. As we noted in the Methods section in reference to S21 Fig, we found that the genetic algorithm was not sensitive to changes to the starting random seeds, and therefore the algorithm is not sensitive to changes to the initial sequence population. We have clarified this issue with reference to S21 Fig.

Reviewer #2:

This manuscript investigates whether integrating different (deep learning) models and objective functions leads to improved computational protein sequence design. By leveraging a Genetic Algorithm as the optimization framework, AlphaFold2 and ProteinMPNN estimated confidence scores are used to define the objective space, and a mutation operator based on ESM-1v and ProteinMPNN to rank and then redesign the least favorable positions. Using the foldswitching protein RfaH as a case study, the authors demonstrate that the proposed evolutionary multiobjective optimization approach improves upon off-the-shelf application of ProteinMPNN. The manuscript is well-written and easy to follow. The experimental design is well-reasoned and justified. I particularly appreciate the in-depth analyses of the results presented and the thoughtful discussion.

We thank the reviewer for their positive comments.

I have couple of suggestions for the authors to consider:

1.) One advantage of the proposed approach over the existing Monte Carlo sampling methods to invert models such as AlphaFold2 for protein design is to that it minimizes the generation of "adversarial" sequences having poor biophysical properties that nevertheless optimize the objective functions. While such a prospect seems reasonable in theory since an adversarial sequence can be propagated only if it is overfitted to by all models, is it practically the case? Can the authors perform some additional experiments to empirically demonstrate that the proposed approach does in fact minimize the generation of adversarial sequences over some of the existing Monte Carlo sampling methods based on inverted AlphaFold2?

We believe that the results from the RfaH GA[RND,RND,AF2Rank] setup are an example of adversarial sequences that can be produced by a simple inversion of AlphaFold2. These sequences, especially at mutation rates 0.1 and 0.3, exhibit minimal native sequence recovery (Fig 2, second column) and high per-position sequence entropy (S5 Fig, first column) that approaches $\ln 20 \approx 2.996$, which is the entropy for a discrete uniform distribution over the allowed 20 standard amino acid types (i.e., the per-position amino acid distribution is close to random guessing). At the same time, these sequences have good Pareto fronts in the AF2Rank objective space that overlaps with the scores of the WT sequence (see the new S6 Fig). As such, these results can be considered an example of adversarial sequences. The fact that the proposed setups such as GA[ESM,pMPNN,pMPNN] and GA[ESM,pMPNN,AF2Rank] have substantially lower sequence entropy (S5 Fig, second and third column) and higher native sequence recovery (Fig 2, fifth and sixth columns) indicate that these setups do not suffer from the same pathology.

We have updated the Results and Discussion sections to highlight and draw a closer connection between the results of the GA[RND,RND,AF2Rank] setup and the concept of adversarial sequences. We have also updated S5 Fig (previously S8 Fig) to show the theoretical upper bound on the sequence entropy distribution to better emphasize the high sequence entropy generated by GA[RND,RND,AF2Rank].

2.) The authors used a baseline custom mutation operator called “Random” (or “RND”) in which each designable position is considered for redesign with a probability controlled by the mutation rate (μ); if no position is chosen after this procedure, a single designable position is picked randomly. Can the authors slightly modify it to come up with an additional baseline operator which can be named “Random_Bin” (or “RND_BIN”) in which k positions randomly chosen are selected for redesign, where k is either 1 or a random number drawn from a binomial distribution Bin(n, μ), whichever is larger; here, n is the total number of designable positions. It may be interesting to include a comparison between “RND_BIN” and “ESM”.

The RND_BIN mutation operator suggested by the reviewer appears to be identical to the RND mutation operator implemented in our work. A random variable following a binomial distribution Bin(n, μ) can be interpreted as the number of successes (i.e., k) in a sequence of n independent, binary experiments/trials, with the success probability given by μ. It thus follows that, by independently considering each designable position for redesign (i.e., a binary trial) with a mutation rate (i.e., success probability) of μ, the number of positions selected for redesign (i.e., k), out of the sequence of n designable positions, is a random variable following the distribution Bin(n, μ).

To demonstrate this argument numerically, the following histogram shows the distribution of k in a numerical simulation of the RND mutation operator repeated over 1,000 times, with n = 100 and μ = 0.3, as well as the probability mass function (PMF) of the corresponding binomial distribution Bin(n, μ), represented by the blue points. The procedure underlying the RND mutation operator converges to a binomial random variable.

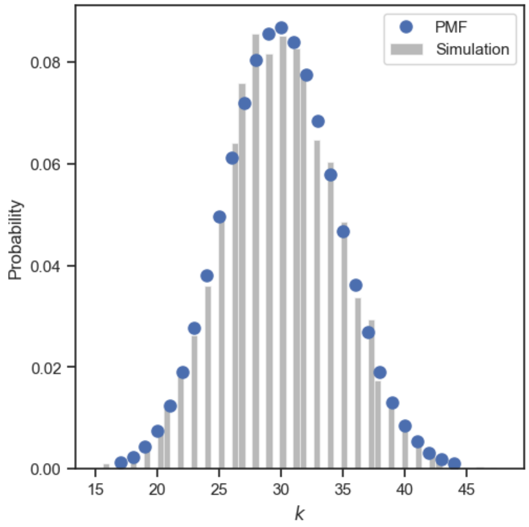

We added additional language in the Methods section to point out the connection between the RND mutation operator and binomial distributions.

We thank both reviewers and the editor for their consideration and hope the manuscript is now suitable for publication in PLoS Computational Biology.

Lu Hong & Tanja Kortemme
